# Supplementary material for: Glutaredoxin Interacts with GR and AhpC to Enhance Low-Temperature Tolerance of Antarctic Psychrophile Psychrobacter sp. ANT206
Source: Int J Mol Sci. 2022 Jan 24;23(3):1313. doi: 10.3390/ijms23031313 (PMC8836231; doi:10.3390/ijms23031313)
Supplement: Supplementary file 1 [file ijms-23-01313-s001.zip › ijms-1567984 - supplementary.pdf]

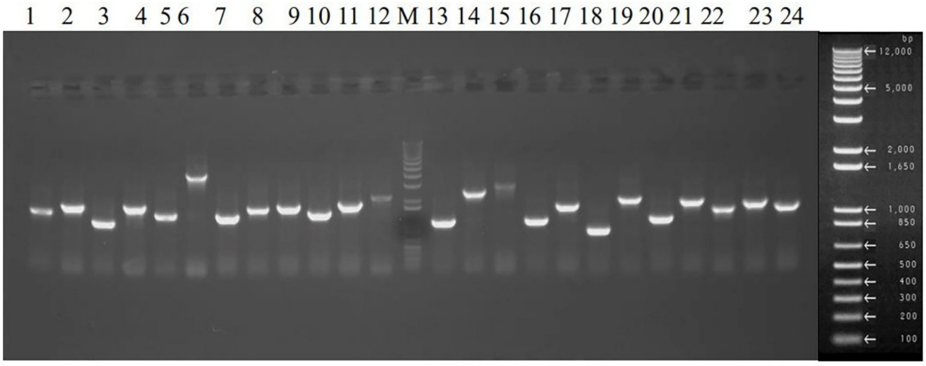

Figure S1: Electrophoresis detection of the inserted fragments in cDNA Library. M, DL12000 DNA marker; 1-24, the PCR amplified fragments of randomly selected colonies.
